# Melatonin Potentiates Exercise-Induced Increases in Skeletal Muscle PGC-1*α* and Optimizes Glycogen Replenishment

**DOI:** 10.3389/fphys.2022.803126

**Published:** 2022-04-26

**Authors:** Vinícius Silva Faria, Fúlvia Barros Manchado-Gobatto, Pedro Paulo Menezes Scariot, Alessandro Moura Zagatto, Wladimir Rafael Beck

**Affiliations:** ^1^ Laboratory of Endocrine Physiology and Physical Exercise, Department of Physiological Sciences, Federal University of São Carlos—UFSCar, São Carlos, Brazil; ^2^ Laboratory of Applied Sport Physiology, School of Applied Sciences, University of Campinas—UNICAMP, Limeira, Brazil; ^3^ Laboratory of Physiology and Sports Performance, Department of Physical Education, School of Science—Bauru Campus, São Paulo State University—UNESP, Bauru, Brazil

**Keywords:** peroxisome proliferator-activated receptor-γ coactivator 1*α* (PGC-1*α*), nuclear respiratory factor 1 (NRF-1), energy metabolism (MeSH ID: D004734), aerobic exercise, N-acetyl-5-methoxytryptamine, recovery, ergogenic aid, skeletal muscle tissue

## Abstract

Compelling evidence has demonstrated the effect of melatonin on exhaustive exercise tolerance and its modulatory role in muscle energy substrates at the end of exercise. In line with this, PGC-1*α* and NRF-1 also seem to act on physical exercise tolerance and metabolic recovery after exercise. However, the literature still lacks reports on these proteins after exercise until exhaustion for animals treated with melatonin. Thus, the aim of the current study was to determine the effects of acute melatonin administration on muscle PGC-1*α* and NRF-1, and its modulatory role in glycogen and triglyceride contents in rats subjected to exhaustive swimming exercise at an intensity corresponding to the anaerobic lactacidemic threshold (iLAn). In a randomized controlled trial design, thirty-nine Wistar rats were allocated into four groups: control (CG = 10), rats treated with melatonin (MG = 9), rats submitted to exercise (EXG = 10), and rats treated with melatonin and submitted to exercise (MEXG = 10). Forty-eight hours after the graded exercise test, the animals received melatonin (10 mg/kg) or vehicles 30 min prior to time to exhaustion test in the iLAn (*t*lim). Three hours after *t*lim the animals were euthanized, followed by muscle collection for specific analyses: soleus muscles for immunofluorescence, gluteus maximus, red and white gastrocnemius for the assessment of glycogen and triglyceride contents, and liver for the measurement of glycogen content. Student t-test for independent samples, two-way ANOVA, and Newman keuls post hoc test were used. MEXG swam 120.3% more than animals treated with vehicle (EXG; *p* < 0.01). PGC-1*α* and NRF-1 were higher in MEXG with respect to the CG (*p* < 0.05); however, only PGC-1*α* was higher for MEXG when compared to EXG. Melatonin reduced the triglyceride content in gluteus maximus, red and white gastrocnemius (*F* = 6.66, *F* = 4.51, and *F* = 6.02, *p* < 0.05). The glycogen content in red gastrocnemius was higher in MEXG than in CG (*p* = 0.01), but not in EXG (*p* > 0.05). In conclusion, melatonin was found to enhance exercise tolerance, potentiate exercise-mediated increases in PGC-1*α*, decrease muscle triglyceride content and increase muscle glycogen 3 h after exhaustive exercise, rapidly providing a better cellular metabolic environment for future efforts.

## 1 Introduction

Melatonin (N-acetyl-5-methoxytryptamine) is considered an indoleamine with amphiphilic characteristics (Amaral et al., 2019). Although it is mainly produced by the pineal gland and directly released into the blood or cerebrospinal fluid, melatonin is also found in several extra pineal tissues, including the brain, retina, liver, skeletal muscle, and so on (Acuña-Castroviejo et al., 2014). Substantial evidence has confirmed the regulatory role of melatonin in circadian and seasonal rhythms (Reiter et al., 2010; Brzezinski et al., 2021; Pevet et al., 2021; Hardeland, 2022), antioxidant (Rodriguez et al. al, 2004; Manchester et al., 2015; Reiter et al., 2016; Ortiz-Franco et al., 2017; Galano et al., 2018; Chitimus et al., 2020; Kruk et al., 2021) and anti-inflammatory effects (Mauriz et al., 2013) among others. In addition to these benefits, our research group has demonstrated the ergogenic effect of melatonin in the increasing exhaustive aerobic exercise tolerance of nocturnal animals (Beck et al., 2015A; Beck et al., 2016; Faria et al., 2021), whereas the effect of melatonin on metabolic recovery after physical exercise is less reported. Specifically, the role of melatonin in muscle glycogen and triglyceride contents few hours after exercise is not fully understood. Additionally, little attention has been given to the role of melatonin administration on PGC-1*α* and NRF-1, which are considered representatives of the aerobic energy metabolism.

In this scenario, we sought to study proteins related to aerobic adaptations that could improve the mitochondrial capacity and possibly modulate the content of energy substrates in the skeletal muscle tissue after an exercise session. Regarding the proteins PGC-1*α* and NRF-1, the peroxisome proliferator-activated receptor-*γ* coactivator 1*α* (PGC-1*α*) is a transcriptional coactivator that interacts with nuclear respiratory factors 1 and 2 (NRF-1 and NRF-2) (Lira et al., 2010; Islam et al., 2018; Islam et al., 2020) to stimulate the mitochondrial biogenesis and function (Bonen, 2009; Seebacher and Glanville, 2010; Islam et al., 2018; Islam et al., 2020). In addition, PGC-1*α* seems to act on the energy metabolism through glycogen content increase (Wende et al., 2007; Wong et al., 2015) and fatty acid oxidation (Wong et al., 2015), with a concomitant enhancement in exhaustive exercise tolerance (Tadaishi et al., 2011; Wong et al., 2015). However, in an opposite scenario of high PGC-1*α*, there are evidences showing a lower endurance performance in skeletal muscle-specific PGC-1*α* knockout (PGC-1*α* MKO) (Handschin et al., 2007) and whole-body PGC-1*α* knockout animals (PGC-1*α* KO) (Leone et al., 2005). Although it is well established that a single bout of exercise is able to increase content and/or expression of PGC-1*α* (Wright et al., 2007; Ikeda et al., 2008; Seebacher and Glanville, 2010; Fujimoto et al., 2011; Shute et al., 2018) and NRF-1 (Murakami et al., 1998; Seebacher and Glanville, 2010; Daussin et al., 2012), reports on these proteins after exercise until exhaustion are lacking for animals treated with melatonin.

Based on the aforementioned assumptions, the literature demonstrates a carbohydrate dependence during prolonged exercise (Leckey et al., 2016; Burke and Hawley, 2018; Hargreaves and Spriet, 2020), establishing that its reduction is a limiting factor for performance (Krssak et al., 2000). Hence, a rapid glycogen repletion following a bout of exhaustive exercise is an important adaptative response to prepare the muscle for subsequent efforts (Wende et al., 2007), at least from a bioenergetic point of view. Moreover, to produce a better exercise training strategy it is essential to understand the beneficial effects of a single bout of exercise since training adaptations reflect the accumulation of beneficial physiological functions produced from acute exercise (Park et al., 2021). Even though some studies suggest that melatonin exerts a modulatory role in muscle energy substrates immediately at the end of exercise (Mazepa et al., 2000; Sanchez-Campos et al., 2001), decreasing carbohydrate utilization and increasing lipid utilization (Mazepa et al., 2000), no study has investigated the effect of melatonin on the recovery of muscle glycogen and triglyceride contents at later times after an exhaustive bout of exercise at an individual and objective intensity. Therefore, the current study aims to determine the effects of acute administration of melatonin on exercise tolerance, glycogen and/or triglyceride contents in the skeletal muscle and liver, as well as PGC-1*α* and NRF-1 expressions in the skeletal muscle of control rats (non-exercised) and rats subjected to exhaustive swimming exercise at an intensity corresponding to the anaerobic lactacidemic threshold (iLAn). We hypothesize that acute melatonin administration increases PGC-1*α*, NRF-1, and muscle glycogen content, in addition to reducing muscle triglyceride content in exercised skeletal muscle, consequently providing a better cellular environment for future efforts.

## 2 Materials and Methods

### 2.1 Animals and Environmental Conditions

Forty young male Wistar rats (45 days old at arrival; weighing between 120 and 150 g) were provided by the Central Animal Facility of the Federal University of São Carlos—UFSCar (Brazil). The animals were housed in controlled environmental conditions: temperature (22 ± 2°C), relative humidity (between 45 and 55%), noise (<85 dB), and photoperiod (10:14 h light/dark cycle), as suggest by the guidelines for the housing of rats in scientific institutions (ARRP Guideline 20). Animals (4–5 per cage) received commercial chow and filtered water *ad libitum*. As albino animals are easily affected by phototoxic retinopathy (ARRP, 2007; Castelhano-Carlos and Baumans, 2009), a stressful condition capable of generating undesirable interference in experiments, especially those involved with melatonin and circadian rhythm, incandescent lamps (Philips, model Soft, 100 W, 2700 K; 565–590 nm; 60 lux, measured by a lux meter) were used during the 10-hour light cycle. To carry out experimental interventions with the rats during the dark cycle (nighttime: 4:00 p.m. to 6:00 a.m.), reflectors were installed around a red filter (ROSCO, model # fire19; > 600 nm; < 15 lux) (Beck and Gobatto, 2013; Beck et al., 2015B; Beck et al., 2016; Faria et al., 2021). Such a luminous scenario makes it possible to prevent the relevant influence of light on the activity of N-acetyltransferase in the pineal gland (Sun et al., 1993), an extremely important enzyme for melatonin biosynthesis. All experimental procedures were conducted in accordance with the Ethical Principles in Animal Research (ARRIVE guidelines 2.0), adopted by the Brazilian College of Animal Experimentation (COBEA, Brazil), and were approved by the Ethics Committee on Animal Use (CEUA) of the Federal University of São Carlos—UFSCar (São Paulo, Brazil) under protocol no. 9144181218.

### 2.2 Experimental Design

In a randomized controlled trial design, the rats (*n* = 39) were divided into four groups: control (CG: *n* = 10), treated with melatonin (MG: *n* = 9), submitted to exercise (EXG: *n* = 10), and treated with melatonin and submitted to exercise (MEXG: *n* = 10). These groups originally numbered 10 animals each, however, throughout the experiment we lost one of them with no defined cause. The animals in the CG and EXG received vehicle solution (ethanol and NaCl, 0.9%), while those belonging to the MG and MEXG received melatonin (10 mg/kg) 30 min before the time to exhaustion test, being euthanized 3 h after the end of the exercise session.

### 2.3 Adaptation to Aquatic Environment and Swimming

After environmental adaptation (from 76 to 89 days old) all rats were adapted to aquatic environment and swimming exercise, considering a protocol adapted from Lima et al. (2017). Initially, the animals were submitted to the aquatic environment in shallow water (10 cm) for 5 min, with increments of 5 min per day for 3 days. Then, the rats were exposed to the swimming exercise protocol in deep water (80 cm) for 2 min in 2-minute increments per day for 7 days. Subsequently, the animals were submitted to swimming exercise in deep water (80 cm) with a load weight of 3% of body mass (attached to the animal’s back) for 5 min, with increments of 5 min per day for 4 days. The animals were introduced to individual swimming protocols in cylindrical and opaque tanks—height: 100 cm (water depth: 80 cm), diameter: 30 cm, and water temperature: 31 ± 1°C, following the guidelines of the American Physiological Society (APS, 2006).

### 2.4 Graded Exercise Test

At 90 days old, all animals were subjected to the graded exercise test (GXT) to determine the exercise intensity corresponding to the individual anaerobic lactacidemic threshold (iLAn). According Beck et al. (2015B), iLAn is found when a disproportionate increase in the concentration of blood lactate is observed with respect to proportional increases in the intensity (imposed through loads corresponding to % of the body mass of each animal), here denominated as the anaerobic lactacidemic threshold. Thus, the animals were subjected to 5-min stages with overloads corresponding to 4.0, 4.5, 5.0, 5.5, 6.0, 6.5, and 7.0% of the body mass (% BM) attached to the animal’s back through elastic strap. Each stage was separated by 30-second intervals in which blood samples (25 µL) were collected from the tip of the animals’ tail and then stored (4°C) to determine lactatemia. The blood lactate concentration was plotted against exercise intensity on a scatter plot, and any change in the blood lactate concentration was identified by visual inspection, as previously described by Matsumoto et al. (1999). Then, two linear regressions were constructed following the break point, and the intersection of these linear regressions interpolated to the *X* axis was used to define the intensity corresponding to the anaerobic lactacidemic threshold (Beck et al., 2015B). The interpolation to *Y* axis corresponded to the blood lactate concentration in the iLAn.

### 2.5 Melatonin Administration

Melatonin (Sigma Aldrich Chemical Corporation; St Louis, MO, United States; M-5250, > 98%) was dissolved in ethanol (< 0.1%) and diluted in saline (0.9% NaCl) for administration at 10 mg/kg (Beck et al., 2015A; Beck et al., 2016; Faria et al., 2021). The preparation was performed prior to its use and stored in an amber bottle wrapped in aluminum foil. Its administration was made intraperitoneally 30 min prior to the time to exhaustion test.

### 2.6 Time to Exhaustion Test (*t*lim)

**FIGURE 1:**
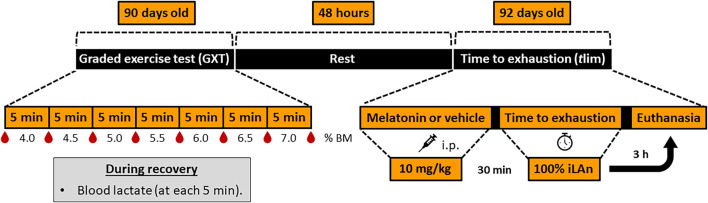
Timeline of events of animals aged from 90 to 92 days old. After adaptation to aquatic environment procedures, at 90 days old all animals were subjected to graded exercise test (GXT) to determine the intensity of exercise corresponding to the individual anaerobic lactacidemic threshold (iLAn). During the GXT, the stages were separated by 30-second intervals in which blood samples (25 µL) were collected. Afterwards, the animals were submitted to the swimming exercise with increments of 0.5% of body mass (% BM) until exhaustion. Forty-eight hours later (rest period), the animals received melatonin (MG and MEXG: 10 mg/kg) or vehicle (CG and EXG: NaCl 0.09%), and after 30 min the rats from EXG and MEXG were submitted to the time to exhaustion test (*t*lim) at 100% of iLAn. After 3 h the animals were euthanized. h, hours; min, minutes; i.p., intraperitoneal.

At 92 days old, 30 min after receiving melatonin (MG and MEXG) or vehicle (CG and EXG) the animals from EXG and MEXG were submitted to swimming exercise until exhaustion in the iLAn, the so-called time to exhaustion test (*t*lim). Then, the animals were introduced to the individual swimming protocol, and the time to exhaustion was recorded. The criterion for identifying the animal’s exhaustion was standardized according to Beck and Gobatto (2013). To this end, the swimming behavior was analyzed in order to observe the execution of vigorous efforts without success in returning to the surface for a period of 15 s. The exhaustion was established by consensus of two experienced observers considering the above criteria. The timeline of events of animals aged from 90 to 92 days old is detailed in Figure 1.

### 2.7 Euthanasia, Obtention and Processing of Biological Materials

All animals were euthanized 3 h after the end of the experimental procedures by decapitation in agreement with the guidelines of the American Veterinary Medical Association (2013). Then, the skeletal muscle tissue (soleus, gluteus maximus, red and white portion of gastrocnemius), and the liver were collected, immediately frozen in liquid nitrogen and stored at −80°C for further analyses.

#### 2.7.1 Determination of Blood Lactate Concentration by an Enzymatic Assay

During the GXT, blood samples (25 μL) were collected from the animals’ tails in heparinized and calibrated glass capillaries. Samples were placed inside plastic tubes (1.5 ml) containing 400 μL of trichloroacetic acid (4%), mixed and stored at 4°C. After stirring and centrifugation (3,000 rpm for 3 min), 50 µL of supernatant was extracted and transferred to a 96-well microplate, where 250 µL of reactive solution prepared for immediate use were added. This reactive solution was comprised of glycine, EDTA, and hydrazine 25%, and after pH adjustment to 9.45, *β*- nicotinamide adenine dinucleotide (NAD), and L-lactic dehydrogenase bovine heart (LDH) were incorporated to the resulting solution. After an incubation period of 20 min at 37°C, the samples were subjected to spectrophotometric measurements (Spectramax i3, Molecular Devices; San José, CA, United States) at 340 nm to compare the sample values to a standard curve constructed from a serial dilution of 1–15 mmol/L of L-Lactate.

#### 2.7.2 Determination of Glycogen Content

The glycogen content within the skeletal muscle (gluteus maximus, red and white portion of gastrocnemius) and the liver was determined as described in Dubois et al. (1956). Both skeletal muscle (250 mg) and liver (500 mg) were firstly immersed in potassium hydroxide (30%; Êxodo Científica; Sumaré, SP, Brazil), and then mixed with saturated sodium sulfate solution (20 μL; Dinâmica Química Contemporânea Ltda; Indaiatuba, SP, Brazil) and ethanol [70%] for glycogen precipitation. The samples were homogenized with phenol (10 μL; Êxodo Científica; Sumaré, SP, Brazil) and sulfuric acid (2 ml; Dinâmica Química Contemporânea Ltda; Indaiatuba, SP, Brazil), and heated in water bath for 5 min (85°C). Finally, the absorbance was measured on a spectrophotometer (Hach Company, Loveland, Colo, United States; 490 nm), and the glycogen content was calculated using a calibration glucose curve.

#### 2.7.3 Determination of Triglyceride Content

To determine the triglyceride content, the skeletal muscle (100–200 mg; gluteus maximus, red and white portion of gastrocnemius) was placed inside plastic tubes (1.5 ml) containing Triton X-100 [1%] at the same proportions (200 mg of tissue to 1 ml of Triton). Next, the samples were homogenized using magnetic bars (5 × 3 mm) overnight (4°C). After this period, the samples were centrifuged (4,000 rpm for 10 min), and 10 µL of the supernatant was extracted, pipetted onto a 96-well microplate in a mixture with the kit reagent (200 μL; LaborLab; Guarulhos, SP, Brazil) and incubated for 20 min (25°C). The triglyceride absorbance was performed on a spectrophotometer (SpectraMax i3, Molecular Devices; San José, CA, United States) at 505 nm, according to the kit’s instructions.

#### 2.7.4 Histology and Immunofluorescence

Immediately after euthanasia, the soleus muscle was dusted in talc, frozen in liquid nitrogen, and stored frozen at −80°C. Afterwards, glass slides (26 × 76 mm) were prepared by sectioning the muscles (6 μm) using a cryostat (Leica CM 1850 UV) at −25°C. The sections were stained by Hematoxilin-Eosin (H&E, MERCK, Darmstadt, Germany) to identify any morphological alterations in tissue through a light microscope.

Immunofluorescence was applied to quantify NRF-1 and PGC-1*α*. The slides with frozen sections were incubated in a mix of primary anti-mouse monoclonal antibodies for NRF-1 (dilution 1:500; Santa Cruz Biotechnology, INC.; Dallas, Texas, United States) or PGC-1*α* (dilution 1:50; Santa Cruz Biotechnology, INC.; Dallas, Texas, United States), conjugated with anti-rabbit laminin (dilution 1:200; Abcam; Ab11575; Cambridge, United kingdom) diluted in 1% BSA (Bovine Serum Albumin—Sigma Aldrich Chemical Corporation, St Louis, MO, United States) for 45 min at 37°C. Then, the slides were washed (3 cycles of 5 min) in PBS solution and incubated in a mix of secondary antibodies: Alexa 488 IgG^1^ to mark NRF-1 in green color (dilution 1:1000; Jackson ImmunoResearch, Laboratories, INC.; West Grove, PA, United States) or Alexa Fluor 647 IgG_2a_ to mark PGC-1*α* in red color (dilution 1:1000; Santa Cruz Biotechnology, INC.; Dallas, Texas, United States), in combination with Alexa Fluor 647 IgG (dilution 1:200; Invitrogen; Carlsbad, California, United States) to mark laminin with a red color or Alexa Fluor 488 IgG to mark laminin with a green color (dilution 1:200; Invitrogen; Carlsbad, California, United States) for 35 min at 37°C. The sections were washed (3 cycles of 5 min) in PBS solution and mounted with FluoroQuest^TM^ Mounting Medium (AAT Bioquest^®^, INC., Sunnyvale, CA, United States).

The slides were analyzed with ImageXpress^®^ Micro (Molecular Devices; San José, CA, United States) using an objective lens with magnification of 20× and specific filters for NRF-1 (FITC—1,200 ms exposure), PGC-1*α* (Cy5—2000 ms exposure), and laminin (FITC and Cy5—100 ms exposure). The integrated density of the fluorescence intensity of NRF-1 and PGC-1*α* was quantified in five distinct and random fields (height: 220 and width: 220) by ImageJ 1.52a software (National Institutes of Health, United States), followed by an individual analysis of the images. The mean value of the proteins in each sample was calculated and plotted in a graph.

### 2.8 Statistical Analysis

A priori power analysis was determined by G*Power 3.1.9.4 software, and it was calculated that a sample size of 40 (10 rats per group) would be required using a two-way ANOVA test at the 5% level of significance with power around 0.77, assuming an effect size of 0.5. The data were presented as a mean ± standard error of the mean. Normality and homogeneity were verified with the Shapiro-Wilk and Levene tests, respectively. When appropriate, outliers were excluded. Time to exhaustion was analyzed through the *t*-test for independent samples comparing exercised animals treated with melatonin (MEXG) versus exercised animals treated with vehicle (EXG). One-way analysis of variance (ANOVA) was used to compare the four groups with regard to the variables obtained from the graded exercise test (iLAn and lactacidemia at this intensity). Two-way ANOVA was applied to analyze other parameters regarding the effects of melatonin (melatonin vs. vehicle) and exercise (exercised vs. non-exercised). When appropriate, the Newman-Keuls post hoc test was used. A significance level of 5% and Statistica 7.0 (StatSoft, Inc.; Tulsa, OK, United States) were used for all analyses. Effect size analysis (ES) and confidence interval (CI) were used as complementary tests. The thresholds for small, moderate, and large effects were 0.20, 0.50, and 0.80, respectively. ES was determined according to Cohen (1988).

## 3 Results

With regard to the variables obtained from the graded exercise test (GXT), no considerable differences were found among the groups in relation to iLAn (CG: 5.41 ± 0.29 (CI = 4.71–6.11), EXG: 5.32 ± 0.17 (CI = 4.92–5.73), MG: 5.51 ± 0.23 (CI = 4.90–6.13), and MEXG: 5.46 ± 0.17 (CI = 5.06–5.85) %BM; *F* = 0.13, *p* = 0.94) and lactacidemia at iLAn (CG: 4.08 ± 0.29 (CI = 3.26–4.89), EXG: 3.93 ± 0.41 (CI = 2.99–4.88), MG: 3.56 ± 0.41 (CI = 2.59–4.52), and MEXG: 4.02 ± 0.22 (CI = 3.52–4.53) mM; *F* = 0.41, *p* = 0.74). Regarding *t*lim, the animals treated with melatonin (MEXG: 105.31 ± 22.89 min; CI = 60.44–150.19) swam 120.3% more than animals treated with vehicle (EXG: 47.81 ± 10.38 min; CI = 27.46–68.15; *p* < 0.01, ES: 1.17).

**FIGURE 2:**
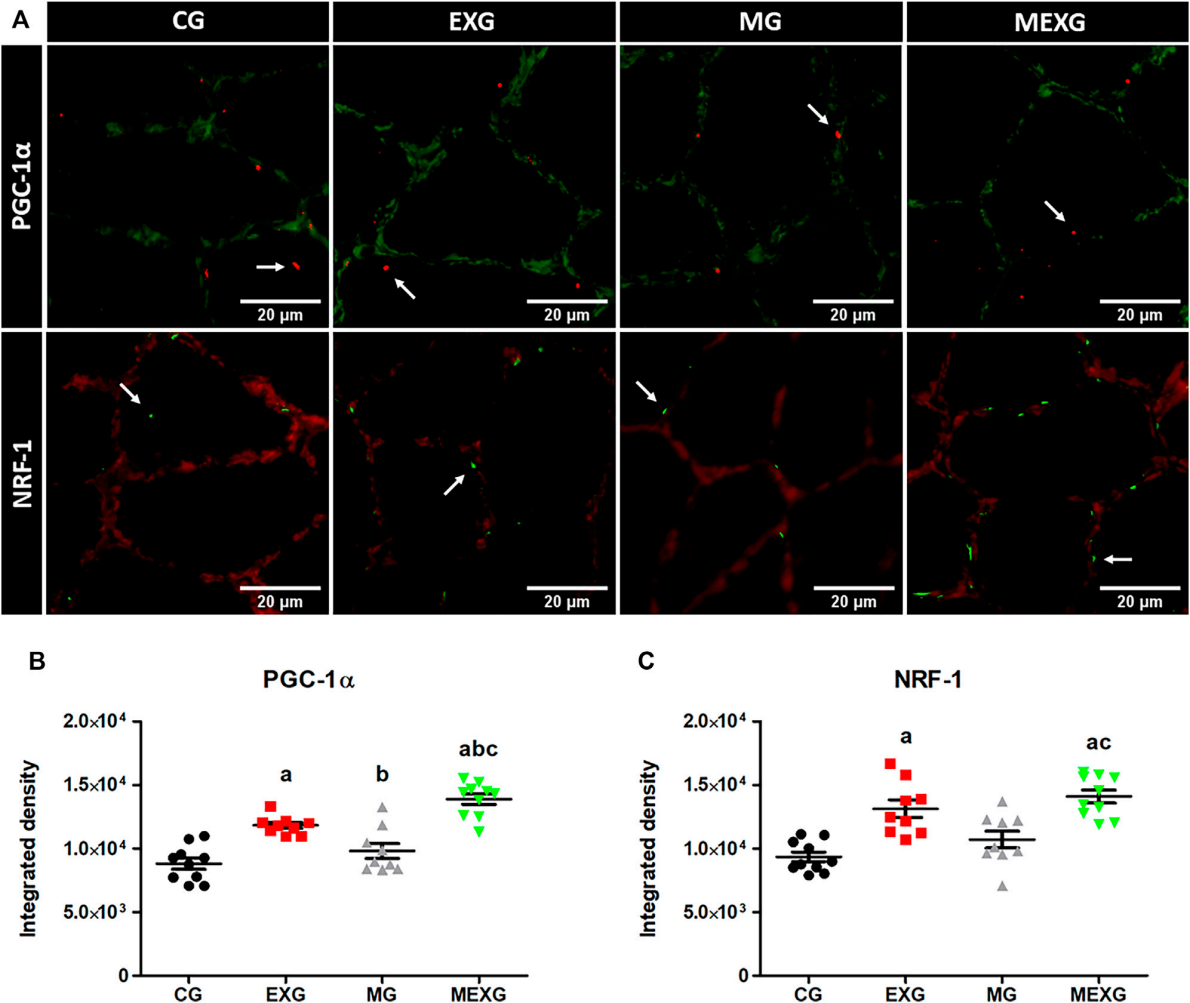
NRF-1 and PGC-1*α* in skeletal muscles. Representative samples of laminin (green) with PGC-1*α* (red) in the soleus skeletal muscle with immunofluorescence (height: 350 and width: 350) **(A)**. Representative samples of laminin (red) with NRF-1 (green) in the soleus skeletal muscle **(A)** with immunofluorescence in the control (CG), rats treated with melatonin (MG), rats submitted to exercise (EXG), and rats treated with melatonin and submitted to exercise (MEXG). The white arrows indicate NRF-1 and PGC-1*α* in the soleus skeletal muscle. The graphs represent the means and standard errors of NRF-1 **(B)** and PGC-1*α* **(C)**. ^a^ *p* < 0.05 with respect to CG; ^b^ p < 0.05 with respect to EXG; ^c^ *p* < 0.05 with respect to MG for the same parameter. For illustration, an objective lens = 20x was used; bars = 20 µm.

Exercise and melatonin increased PGC-1*α* (*F* = 64.64, *p* < 0.01 and *F* = 12.00, *p* < 0.01) and NRF-1 (*F* = 39.81, *p* < 0.01 and *F* = 4.20, *p* < 0.05) (Figure 2). Large effects on PGC-1*α* were obtained when comparing CG vs. EXG (*p* < 0.01, ES: 2.84; EXG > CG), EXG vs. MG (*p* < 0.01, ES: 1.64; EXG > MG), CG vs. MEXG (*p* < 0.01, ES: 3.69; MEXG > CT), EXG vs. MEXG (*p* < 0.01, ES: 2.00; MEXG > EXG), and MG vs. MEXG (*p* < 0.01, ES: 2.64; MEXG > MG). Mean ± SEM and confidence interval values on PGC-1*α* for CG (8,811.76 ± 444.63; CI = 7,805.92–9,917.60), EXG (11,824.22 ± 238.15; CI = 11,275.03–12,373.42), MG (9,809.93 ± 582.68; CI = 8,466.26–11,153.60), and MEXG (13,877.66 ± 423.28; CI = 12,920.12–14,835.20). Likewise, large effects on NRF-1 were found when comparing CG vs. EXG (*p* < 0.01, ES: 2.84; EXG > CG), CG vs. MEXG (*p* < 0.01, ES: 3.69; MEXG > CG), and MG vs. MEXG (*p* < 0.01, ES: 1.87; MEXG > MG). Mean ± SEM and confidence interval on NRF-1 for CG (9,341.66 ± 389.76; CI = 8,459.94–10,223.38), EXG (13,130.80 ± 694.19; CI = 11,529.98–14,731.62), MG (10,711.16 ± 669.48; CI = 9,167.32–12,254.99), and MEXG (14,089.66 ± 508.42; CI = 12,939.53–15,239.79).

**FIGURE 3:**
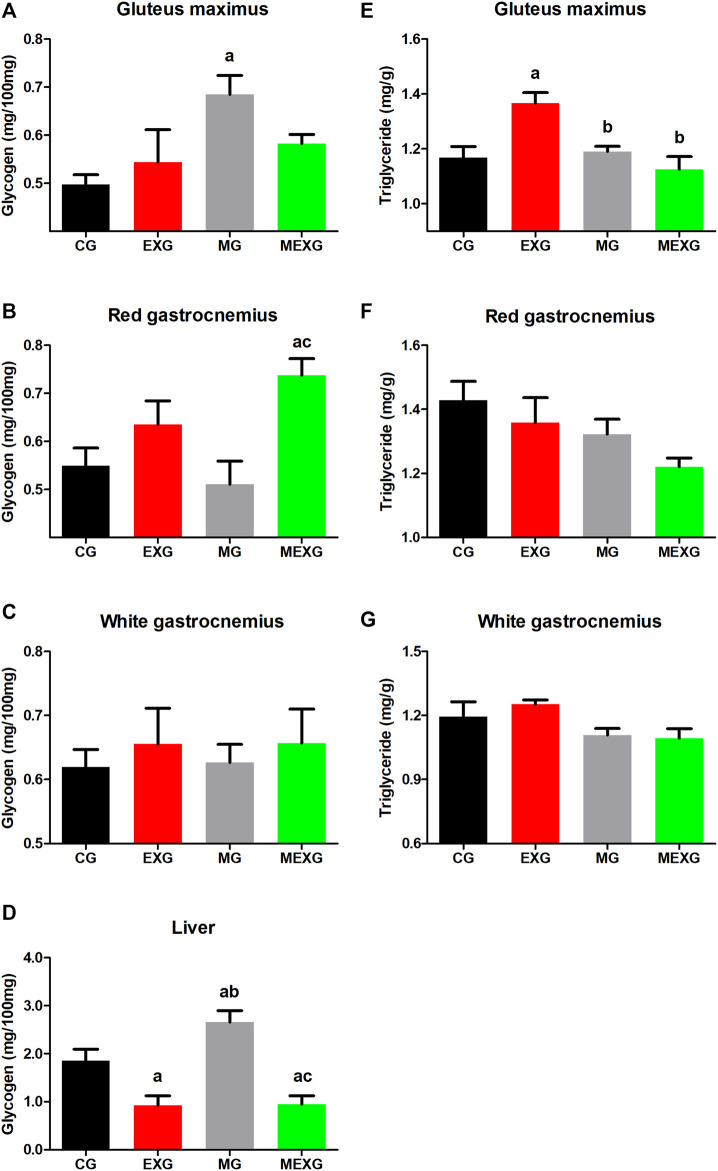
Glycogen content in skeletal muscles and liver, and triglyceride content in skeletal muscles. The graphs represent the means and standard errors of the glycogen content in the gluteus maximus **(A)**, red **(B)** and white **(C)** gastrocnemius and liver **(D)**, and triglyceride content in gluteus maximus **(E)**, red **(F)** and white **(G)** gastrocnemius in control rats (CG), rats treated with melatonin (MG), rats submitted to exercise (EXG), and rats treated with melatonin and submitted to exercise (MEXG). ^a^ *p* < 0.05 with respect to CG; ^b^ *p* < 0.05 with respect to EXG; ^c^ *p* < 0.05 with respect to MG for the same parameter. mg, milligrams; g, grams; min, minutes.

As to glycogen, exercise increased its content in red gastrocnemius (*F* = 13.32, *p* < 0.01) but decreased in liver (*F* = 37.70, *p* < 0.01), while no difference was observed in gluteus maximus and white gastrocnemius (*F* = 0.35, *p* = 0.55 and *F* = 0.56, *p* = 0.45). Furthermore, melatonin increased the glycogen content in gluteus maximus (*F* = 5.71, *p* = 0.02), but did not promote any difference in liver (*F* = 3.59, *p* = 0.06), red and white gastrocnemius (*F* = 0.55, *p* = 0.46 and F < 0.01, *p* = 0.92, respectively) (Figures 3A–D). Large effects were observed on glycogen content in liver (CG vs. EXG (*p* = 0.01, ES: 1.36; CG > EXG), CG vs. MG (*p* = 0.01, ES: 1.10; MG > CG), EXG vs. MG (*p* < 0.01, ES: 2.61; MG > EXG), CG vs. MEXG (*p* < 0.01, ES: 1.41; CG > MEXG), and MG vs. MEXG (*p* < 0.01, ES: 2.73; MG > MEXG)), gluteus maximus (CG vs. MG (*p* = 0.04, ES: 2.13; MG > CG)) and red gastrocnemius (CG vs. MEXG (*p* = 0.01, ES: 1.71; MEXG > CG) and MG vs. MEXG (*p* < 0.01, ES: 1.83; MEXG > MG)). Mean ± SEM and confidence interval on glycogen content for liver: CG (1.85 ± 0.23; CI = 1.31–2.39), EXG (0.92 ± 0.19; CI = 0.48–1.36), MG (2.65 ± 0.23; CI = 2.11–3.20), and MEXG (0.94 ± 0.18; CI = 0.52–1.36); gluteus maximus: CG (0.49 ± 0.01; CI = 0.45–0.54), EXG (0.54 ± 0.06; CI = 0.39–0.69), MG (0.68 ± 0.03; CI = 0.59–0.77), and MEXG (0.58 ± 0.01; CI = 0.53–0.62); red gastrocnemius: CG (0.54 ± 0.03; CI = 0.46–0.63), EXG (0.63 ± 0.04; CI = 0.52–0.74), MG (0.51 ± 0.04; CI = 0.39–0.62), and MEXG (0.73 ± 0.03; CI = 0.65–0.81); and white gastrocnemius: CG (0.61 ± 0.02; CI = 0.55–0.68), EXG (0.65 ± 0.05; CI = 0.52–0.78), MG (0.62 ± 0.02; CI = 0.56–0.69), and MEXG (0.65 ± 0.05; CI = 0.53–0.77).

In relation to triglyceride content, exercise did not cause any change in skeletal muscles, such as gluteus maximus (*F* = 2.51, *p* = 0.12), red and white gastrocnemius (*F* = 2.23, *p* = 0.14 and *F* = 0.18, *p* = 0.66, respectively). On the other hand, melatonin decreased the triglyceride content in gluteus maximus (*F* = 6.66, *p* = 0.01) red and white gastrocnemius (*F* = 4.51, *p* = 0.04 and *F* = 6.02, *p* = 0.02) (Figures 3E–G). Large effects on the triglyceride content in gluteus maximus were noted when comparing CG vs. EXG (*p* < 0.01, ES: 1.63; EXG > CG), EXG vs. MG (*p* < 0.01, ES: 2.22; EXG > MG), and EXG vs. MEXG (*p* < 0.01, ES: 1.84; EXG > MEXG). Mean ± SEM and confidence interval on triglyceride content for gluteus maximus: CG (1.16 ± 0.04; CI = 1.07–1.25), EXG (1.36 ± 0.03; CI = 1.27–1.45), MG (1.19 ± 0.01; CI = 1.14–1.23), and MEXG (1.12 ± 0.04; CI = 1.01–1.23); red gastrocnemius: CG (1.42 ± 0.05; CI = 1.29–1.56), EXG (1.35 ± 0.07; CI = 1.18–1.53), MG (1.32 ± 0.04; CI = 1.21–1.43), and MEXG (1.22 ± 0.02; CI = 1.15–1.28); and white gastrocnemius: CG (1.19 ± 0.07; CI = 1.03–1.35), EXG (1.25 ± 0.02; CI = 1.20–1.29), MG (1.10 ± 0.03; CI = 1.03–1.18), and MEXG (1.09 ± 0.04; CI = 0.98–1.20).

## 4 Discussion

Among the main findings of this study, we can highlight the ability of melatonin to potentiate exercise-mediated increases in PGC-1*α*, to reduce muscle triglyceride content and to increase glycogen content 3 h after exhaustive exercise session, possibly favoring cellular environment for future efforts, thus confirming our initial hypothesis. This is the first study to analyze the acute effect of melatonin administration on PGC-*α* and NRF-1 and its influence on the replenishment of glycogen in rats submitted to an individualized exhaustive exercise session with intensity corresponding to the anaerobic lactacidemic threshold.

To evaluate the beneficial effect of melatonin and exercise on the mitochondrial biogenesis, we quantified PGC-1*α*, a transcriptional coactivator that functions as a master regulator of the mitochondrial biogenesis (Booth et al., 2015). With regard to the effect of melatonin, PGC-1*α* and NRF-1 of the animals treated with melatonin showed an increase compared to those treated with vehicle (*F* = 12.00, *p* < 0.01 and *F* = 4.20, *p* < 0.05, respectively). These effects possibly occurred via melatonin receptors that were found to be present in the skeletal muscle membrane [Ha et al., 2006; (BioGPS, 2021) (http://ds.biogps.org/?dataset=GSE952&gene=114211)]. The literature postulates that melatonin acts through the activation of CAMKII, inducing CREB phosphorylation, and consequently increasing the expression of PGC-1*α* in the skeletal muscle (Teodoro et al., 2014). Such increase leads to the activation of key genes involved in the mitochondrial biogenesis, such as NRF-1 (Jung and Kim, 2014; Islam et al., 2020). Additionally, it seems that the activation pathway of PGC-1*α* is tissue-dependent. In heart tissue, melatonin can act via AMPK-PGC-1*α* to improve the mitochondrial biogenesis (Yu et al., 2017; Qi and Wang, 2020). Therefore, even though a positive effect of melatonin was observed a few hours after acute administration, further studies are still necessary to better understand the pathways involved in the activation of PGC-1*α* in the skeletal muscle of animals treated with melatonin.

Regarding the effect of exercise, the PGC-1*α* and NRF-1 of exercised animals showed higher expression in relation to non-exercised animals (*F* = 64.64, *p* < 0.01 and *F* = 39.81, *p* < 0.01, respectively). It is well known that exercise is one of the main stimuli for PGC-1*α* activation. Thus, according to the literature a single bout of exercise can activate calcium/calmodulin-dependent protein kinase (CaMK), p38 mitogen-activated protein kinase (p38 MAPK), cyclic adenosine monophosphate (cAMP), and phosphorylate AMP activated protein kinase (AMPK), which are the molecular signals responsible for the increase in PGC-1*α* expression (Bonen, 2009; Lira et al., 2010; Perry and Hawley, 2018; Memme et al., 2021). Our results are in accordance with the literature and demonstrate that a single bout of exercise increases the expression and/or content of PGC-1*α* (Wright et al., 2007; Ikeda et al., 2008; Seebacher and Glanville, 2010; Fujimoto et al., 2011; Shute et al., 2018) and NRF-1 (Murakami et al., 1998; Seebacher and Glanville, 2010; Daussin et al., 2012). More importantly, our main finding is that melatonin potentiates the up-regulation of PGC-1*α* expression. Curiously, the PGC-1*α* up-regulation mediated by melatonin was found only in exercised rats (but not in non-exercised animals). These observations suggest that melatonin effects are pronounced during challenging situations. This is in congruence with the findings from previous studies conducted by our group, who reported the effect of melatonin only in the presence of a stressful stimulus, such exhaustive exercise (Beck et al., 2015A). Furthermore, other authors demonstrated interesting effects of melatonin on *in vitro* palmitic acid-induced insulin resistance model or *in vivo* pinealectomized rats, evidencing the increase in the PGC-1*α* expression in both situations (Teodoro et al., 2014). In line with this rationale, melatonin (in the absence of a stressful stimulus of exercise) did not cause any change in PGC-1*α* and NRF-1 when compared to non-exercised animals treated with vehicle (MG vs. CG; *p* > 0.05).

Concerning exercise performance, compelling evidence has shown a significant lower performance in isometric and dynamic muscle endurance, assessed by muscle grip strength test and graded exhaustive running treadmill exercise test in skeletal muscle-specific PGC-1*α* knockout (PGC-1*α* MKO) (Handschin et al., 2007) or whole-body PGC-1*α* knockout mice (PGC-1*α* KO) (Leone et al., 2005) when compared to control animals. According to Leone et al. (2005), the exercise capacity was partly reduced due to abnormalities in the mitochondrial structure of the skeletal muscle and the function of PGC-1*α* KO in animals with lower maximal oxygen consumption (VO_2max_) and fatigue resistance index than control mice (*p* < 0.05).

In an opposite scenario of low PGC-1*α*, there is evidence showing that animals with overexpressed skeletal muscle-specific PGC-1*α* (PGC-1*α* MKC) reached a longer distance, obtained a higher oxygen uptake (VO_2_) (Wong et al., 2015), and peak oxygen consumption (VO_2peak_) (Tadaishi et al., 2011) than control animals during graded maximal exercise test (*p* < 0.05). Therefore, when analyzing our results, we believe that the increase in exhaustion time presented by animals treated with melatonin (MEXG) in comparison with animals treated with vehicle (EXG: *p* < 0.01, ES: 1.17) may have occurred due to two factors: 1) the ability of melatonin to potentiate the effect of muscle contraction on PGC-1*α* (as seen by MEXG with respect to EXG; *p* = 0.002, ES: 2.00), thus allowing greater exercise tolerance. In line with this, Wong et al. (2015) and Tadaishi et al. (2011) demonstrated a significantly positive relationship between exercise tolerance and PGC-1*α*. 2) According to Sanchez-Campos et al. (2001) and Mazepa et al. (2000), the animals treated with melatonin and euthanized approximately 2 h (30 min + time to exhaustion) after melatonin administration obtained a higher liver and/or muscle glycogen content than control animals (*p* < 0.05). A similar behavior was observed in our results, which confirmed the ability of melatonin to increase the glycogen content in animals euthanized approximately 4 h (30 min + time to exhaustion + 3 h) after melatonin administration (as seen by MG compared to CG for gluteus maximus and liver (*p* < 0.05; ES: 2.13 and ES: 1.10, respectively)). Thus, considering that this exercise model (*t*lim) considerably depleted the muscle glycogen content (Beck et al., 2014) and that its absence is a limiting factor for performance (Krssak et al., 2000), we believe that the increase in time to exhaustion (*t*lim) presented by MEXG in comparison with EXG (*p* < 0.01, ES: 1.17) may also have been a result of the increase in the pre-exercise glycogen content. However, there are no studies involving the acute effect of melatonin on the pre-exercise glycogen content (30 min after administration) and its subsequent use during exhaustive exercise (*t*lim). Hence, more studies must be conducted to deepen the understanding of such issue.

Concerning metabolic recovery, the mitochondrial capacity for substrate oxidation in skeletal muscle is the major determinant of performance (Halling et al., 2019), as well as the metabolic recovery after physical exercise, through the replenishment of glycogen. Besides the role in the biogenesis and mitochondrial function, PGC-1*α* and NRF-1 are directly linked to the regulation of energy substrates for the skeletal muscle (Bonen, 2009), resulting in a profound increase in its capacity to use lipid substrate (Wong et al., 2015). We then believe that the increase in PGC-1*α* and NRF-1 led to a lower triglyceride content, as demonstrated by the animals treated with melatonin in relation to those treated with vehicle for all skeletal muscles, such as gluteus maximus (*F* = 6.66, *p* = 0.01), red and white gastrocnemius (*F* = 4.51, *p* = 0.04 and *F* = 6.02, *p* = 0.02). These results are in accordance with the literature, which demonstrated a better lipid profile for animals treated with melatonin, decreasing intramuscular fat deposition by promoting lipolysis and increasing mitochondrial function in porcine intramuscular preadipocytes (Liu et al., 2019), as well as reduced blood triglyceride concentration (Agil et al., 2011; Mendes et al., 2013; Lolei et al., 2019). Considering the reduction in the muscle triglyceride content, a higher glycogen content was expected in gluteus maximus, as demonstrated by the animals treated with melatonin compared with those treated with vehicle (*F* = 5.71, *p* = 0.02).

In relation to glycogen replenishment, it is important to note that the animals treated with melatonin (MEXG) swam 120.3% more than those treated with vehicle (EXG; *p* < 0.05, ES: 1.17), which made us expect a lower content of glycogen, as suggested by the literature (Matsui et al., 2011; Beck et al., 2014). However, when analyzing our results, the animals treated with melatonin and submitted to exercise (MEXG) were statistically equal to those treated with vehicle and submitted to exercised (EXG) in terms of glycogen content in the liver (*p* > 0.05) and the skeletal muscles (gluteus maximus, red and white gastrocnemius (*p* > 0.05)), even though a difference in *t*lim appeared between the groups (MEXG > EXG; *p* < 0.05). Moreover, in the presence of melatonin, there was an overcompensation of the glycogen content in the red gastrocnemius of exercised animals (MEXG; *p* = 0.01, ES: 1.71). However, this did not occur in the absence of melatonin (EXG; *p* > 0.05) in comparison with the control animals (CG). Therefore, we can consider that melatonin accelerates the replenishment of energy substrates, which may have facilitated the increase in glycogen stores.

In this scenario, a rapid glycogen repletion following a bout of exhausting intense exercise is an important response to prepare the muscle for subsequent bouts of activity (Wende et al., 2007), specially for sports with repeated bouts of exercise at the same day or in the following day. Thereby, melatonin seems to optimize the response to exercise since training adaptations reflect the accumulation of beneficial physiological functions produced from single bouts of exercise (Park et al., 2021). Despite the positive results of this study, there are still some limitations that must be addressed: 1) We only focused on the master regulator of the mitochondrial biogenesis and its relationship with NRF-1; however, evaluating other proteins involved in the mitochondrial biogenesis process as well as the PGC-1*α* downstream signals could provide additional information on the mechanism of action of melatonin; 2) we chose the dosage of 10 mg/kg due to the effects of melatonin on time to exhaustion (*t*lim), as previously reported by our research group (Beck et al., 2015A; Beck et al., 2016; Faria et al., 2021); nonetheless, another concentration of melatonin should be tested to achieve a similar effect with lower dosage. Therefore, our findings make it clear that future studies must be conducted in order to deepen the understanding of the importance of melatonin from a physiological point of view.

In summary, the current study highlighted the role of melatonin in the increase of exercise tolerance, exercise-mediated PGC-1*α* and muscle glycogen after exhaustive prolonged exercise, as well as in the decrease of muscle triglyceride content, thus providing a better cellular metabolism environment for future efforts and virtually improving adaptive responses to training.

## 5 Future Perspectives

If confirmed in humans, the outcomes of this study could be useful for athletes who must quickly return to their training or competitive activities; For sure, further studies are needed to elucidate whether such effects occur similarly in humans. In addition, the administration of melatonin in the context of training recovery should be more explored and expanded to other health areas. In this line, melatonin could be useful in treating/avoiding overtraining, a condition in which energy stores as glycogen are chronically low (Fry et al., 1991; Smith, 2000).

## Data Availability

The original contributions presented in the study are included in the article, further inquiries can be directed to the corresponding author.

## Abbreviations

ES, Effect size; GXT, graded exercise test; iLAn, intensity of anaerobic lactacidemic threshold; PGC-1*α* KO, whole-body PGC-1*α* knockout animals; PGC-1*α* MKO, skeletal muscle-specific PGC-1*α* knockout.
